# Cardiovascular Diseases: From Basic Research to Clinical Application—3rd Edition

**DOI:** 10.3390/life16060931

**Published:** 2026-06-01

**Authors:** Cristiana Bustea, Delia Mirela Tit

**Affiliations:** 1 Department of Preclinical Disciplines, Faculty of Medicine and Pharmacy, University of Oradea, 410087 Oradea, Romania; 2 Department of Pharmacy, Faculty of Medicine and Pharmacy, University of Oradea, 410087 Oradea, Romania

The third edition of the Special Issue ‘Cardiovascular Diseases: From Basic Research to Clinical Application’ has brought together a diverse and high-quality body of work reflecting the complexity, heterogeneity, and ongoing evolution of cardiovascular medicine. As Guest Editors, we are particularly encouraged by the broadness of approaches, from molecular and experimental investigations to clinical studies, technological innovations, and illustrative case reports, each contributing to a more integrated understanding of cardiovascular disease.

A central theme of this Special Issue is the exploration of molecular and inflammatory mechanisms underlying cardiovascular disease. In this context, the study by Papanikolaou et al. demonstrated that NLRP3 inflammasome components remain significantly upregulated weeks after acute coronary syndrome, suggesting persistent immune activation and a potential substrate for residual inflammatory risk [1]. Complementing this, the comprehensive review by Zivalj et al. highlighted that microRNAs act as key regulators of cardiac remodeling and heart failure progression, with promising roles as both biomarkers and therapeutic targets, further supported by emerging AI-driven analytical approaches [2]. Micieta et al. showed that interactions between inflammatory markers, autonomic regulation, and adiposity are already present in adolescents and exhibit sex-specific patterns, pointing to early and differential pathways of cardiovascular risk development [3].

Bridging basic science with translational potential, Emir et al. provided compelling experimental evidence that thiamine pyrophosphate, but not thiamine, effectively attenuates hydroxychloroquine-induced cardiomyopathy by restoring oxidative balance and improving biochemical and histopathological parameters [4]. This work underscores the importance of targeted metabolic interventions in drug-induced cardiotoxicity and highlights the need for further translational validation.

Several contributions addressed the complexity of cardiovascular disease in real-world clinical settings, particularly in the presence of comorbidities and heterogeneous pathophysiological mechanisms. Darabont et al. emphasized that hypertension significantly contributes to the development and progression of valvular heart disease through hemodynamic and structural mechanisms, with important implications for prognosis and management [5]. Focusing on coronary pathophysiology, Hung and Hung underscored that coronary artery spasm is a frequently underdiagnosed cause of ischemia in patients with non-obstructive coronary arteries, advocating for comprehensive intracoronary provocation testing to ensure accurate diagnosis and treatment [6]. In a related context, Matei et al. highlighted that coronary artery spasm, both epicardial and microvascular, plays a major role in persistent symptoms and adverse outcomes in patients with type 2 diabetes mellitus, driven by hyperglycemia-induced vascular dysfunction [7].

Advances in interventional cardiology and therapeutic strategies were also clearly featured. Gherasie et al. demonstrated that drug-coated balloons, including both paclitaxel- and sirolimus-based technologies, represent viable alternatives to drug-eluting stents in selected clinical scenarios, particularly in in-stent restenosis and small-vessel disease [8]. In parallel, Soczyńska et al. introduced the DynamX bioadaptor as a novel stent technology capable of restoring physiological vessel function through its unique “uncaging” mechanism, potentially overcoming limitations of conventional stents [9].

Expanding beyond procedural interventions, Gherghin et al. reviewed the emerging role of digital health, showing that AI-enhanced telerehabilitation enables personalized, adaptive, and data-driven post-acute coronary syndrome care, although robust clinical evidence is still needed to support widespread implementation [10].

The inclusion of case reports adds valuable clinical depth by illustrating rare conditions and diagnostic challenges. Bustea et al. described a rare right ventricular myxoma presenting with pulmonary outflow obstruction and embolic risk, successfully managed surgically, while exploring a possible but unconfirmed association with prior malignancy [11]. Similarly, Kitov et al. presented a diagnostically challenging case demonstrating that pregnancy-associated myocardial infarction can mimic peripartum cardiomyopathy, highlighting the essential role of multimodality imaging in achieving diagnostic accuracy in complex peripartum cardiovascular conditions [12].

Taken together, the contributions to this Special Issue highlight the importance of integrating molecular insights, clinical phenotyping, technological innovation, and individualized patient care. As Guest Editors, we would like to express our sincere gratitude to all authors for their excellent contributions, to the reviewers for their rigorous and constructive evaluations, and to the editorial team for their continuous support and professionalism throughout this process.

As cardiovascular diseases remain a leading cause of global morbidity and mortality, continued interdisciplinary research, bridging basic science, clinical practice, and emerging technologies, will be essential to further improve prevention, diagnosis, and treatment. It is my hope that this collection of articles will stimulate future investigations, encourage collaboration, and contribute meaningfully to advancing the field of cardiovascular medicine.
